# Multiplex PCR-Based Detection of Eight Carbapenemase Genes and Their Clinical Characteristics in Urinary Tract Infections

**DOI:** 10.3390/antibiotics15060529

**Published:** 2026-05-22

**Authors:** Nishadi Jayathilaka, Upeksha Kulasekara, Dilini Nakkawita, Dharshan De Silva, Samanmalee Gunasekara, Thamarasi Senaratne

**Affiliations:** 1 Faculty of Graduate Studies, General Sir John Kotelawala Defence University, Colombo 10390, Sri Lanka; shanijayathilaka2@gmail.com (N.J.); u.s.k.upekshasewwandi@gmail.com (U.K.); 2 Department of Paraclinical Sciences, Faculty of Medicine, General Sir John Kotelawala Defence University, Colombo 10390, Sri Lanka; dilininak@kdu.ac.lk (D.N.); dharshan_fom@kdu.ac.lk (D.D.S.); 3 National Cancer Institute, Maharagama 10280, Sri Lanka; 65samanmalee@gmail.com; 4 Department of Medical Laboratory Sciences, Faculty of Allied Health Sciences, General Sir John Kotelawala Defence University, Colombo 10390, Sri Lanka

**Keywords:** carbapenemase genes, carbapenem resistance, molecular characterization, multiplex PCR, Sri Lanka, urinary tract infections (UTIs)

## Abstract

**Background:** The emergence and spread of urinary carbapenem-resistant organisms (CROs) are a major public health concern, particularly in Sri Lanka. Therefore, we aimed to detect and genotypically characterize CROs in urinary tract infections (UTIs) and their clinical outcomes. **Methods:** Urinary CROs were collected from two hospitals in Sri Lanka from January to December, 2023. Among 7640 urine samples, 100 CROs were identified by disk diffusion method, and 99 were detected by BD Pheonix^TM^ automated system. The presence of eight carbapenemase genes; *bla*_KPC_, *bla*_NDM_, *bla*_VIM_, *bla*_IMP_, *bla*_OXA-23_, *bla*_OXA-48_, *bla*_OXA-51_, and *bla*_OXA-58_, among 97 CROs was detected by a multiplex PCR kit. **Results:** Out of 99 urinary CROs, *K. pneumoniae* (33.3%; *n* = 33/97) was the most common species. Among the 97 isolates tested by PCR, a single carbapenemase gene was detected in 35.05% (34/97), while two or more genes co-occurred in 39.18% (38/97). The most frequently identified gene was *bla*_OXA-51_ (47.4%), followed by *bla*_OXA-58_ (41.2%). Most patients (95.74%; *n* = 90/97) showed clinical improvement within seven days of treatment. Among the 93 patients discharged and followed for three months, 74.20% (*n* = 69/93) experienced at least one mild UTI recurrence. A total of 10 patients died during the study period. Of which, four (40%) during hospitalization and six (60%) during follow-up, though none of the deaths were attributed to UTIs. **Conclusions:** *K. pneumoniae*, showed the highest carbapenemase gene diversity. Recurrent UTIs were observed during the follow-up period. Continuous surveillance and implementation of targeted infection control programs are needed to minimize further emergence and spread of carbapenemase genes.

## 1. Introduction

Antibiotic resistance is one of the significant global health challenges of the 21st century, threatening the effective prevention and treatment of an ever-increasing range of infections caused by bacteria. Among bacterial pathogens, Gram-negative bacteria (GNB) have developed complex resistance mechanisms, with carbapenem-resistant organisms (CROs) posing a critical threat due to their ability to hydrolyze last-resort antibiotics such as carbapenems [1,2]. An alarming trend of CROs was observed globally in recent years [2]. The World Health Organization (WHO) published its first list of “antibiotic-resistant priority pathogens”, including carbapenem-resistant Enterobacteriaceae (CRE), carbapenem-resistant *Acinetobacter*, and carbapenem-resistant *Pseudomonas* as the most critical groups [3]. In 2017, 13,100 infections in hospitalized patients were due to CRE, and 1100 deaths were reported in the United States [4]. The mortality and morbidity associated with infections caused by CROs are relatively greater than those associated with infections caused by carbapenem-sensitive pathogens [5,6].

Urinary tract infections (UTIs) have caused a significant impact on healthcare systems worldwide, affecting individuals across all age groups. While most UTIs are typically treated with readily available antibiotics, the rise in carbapenem resistance among uropathogens has further complicated the treatment and management of affected patients [1]. The majority of CREs cause UTIs [7], in which patients with urinary catheters are at a greater risk [4]. The public health implications of UTIs caused by CROs are profound, due to their significant contribution to healthcare-associated infections, community morbidity and broader dissemination of resistance genes [6,7].

The major mechanism of resistance to carbapenem antibiotics is known to involve the production of carbapenemase enzymes [8]. These carbapenemases hydrolyze broad-spectrum β-lactam antibiotics including carbapenems, which are considered the last resort for the treatment of infections caused by multidrug-resistant (MDR) bacteria [7,9,10]. These carbapenemases are categorized into different classes based on their amino acid sequences. Ambler class A and D include serine β-lactamases, whereas class B includes metallo-β-lactamases. These classes were further divided considering carbapenemases within each class. Ambler class A includes *Klebsiella pneumoniae* carbapenemase (*bla*_KPC_), whereas class B includes Verona Integron encoding metallo-β-lactamase (*bla_V_*_IM_), New Delhi metallo-β-lactamase (*bla*_NDM_) and imipenemase (*bla*_IMP_). The Ambler class D includes oxacillinases such as *bla*_OXA-23_, *bla*_OXA-48_, *bla*_OXA-181_, etc. [7,8,10,11]. Variations in the minimum inhibitory concentrations (MICs) of carbapenems occur depending on the type and expression of carbapenemase enzymes present, the type of CRO present, and the presence of other resistance mechanisms [11].

Many carbapenemase-encoding genes originated in India (i.e., *bla*_NDM-1_ and *bla*_OXA-181_), and India is considered a reservoir for most of the carbapenemase genes [12,13]. The study by Tesfa et al. reported the highest prevalence rate of carbapenem-resistant *K. pneumoniae* from India, China, Egypt, Spain and USA, ranging from 15% to 22%, and the lowest prevalence rate from Japan (0.13%). In addition, the highest CRO colonization rate was observed in Asia, mainly in China and India (1.4%), followed by Europe (1.2%), America (0.3%), and Africa (0.07%) indicating that the CRO distribution is not uniform even within a single country [14,15].

Sri Lanka is located at the southern tip of India. Therefore, there is an increased risk of carbapenemase gene dissemination to Sri Lanka via the frequent movement of people between the two countries [16]. Additionally, an increasing trend of CROs has been reported, ranging from 8.3% in 2017 to 35.2% in 2022, posing a substantial challenge in patient management [17,18,19,20,21]. As Sri Lanka is a developing country with a free healthcare system, this alarming carbapenem resistance rate is a crucial concern for the economy [19]. Although data on the epidemiology and characterization of carbapenemase genes were reported a few years ago from Sri Lanka [20,21,22], recent data on UTIs caused by CROs, including their resistance mechanisms and the clinical characteristics of these patients, are lacking. Therefore, the objective of the present study was to detect carbapenemase genes present among urinary isolates that were initially resistant to carbapenem antibiotics by the Clinical Laboratory Standard Institute (CLSI) disk diffusion method, and to assess the clinical characteristics of the respective UTI patients over a period of three months.

## 2. Results

### 2.1. Demographic Characteristics

A total of 7640 urine samples were received by the microbiology laboratories of the University Hospital, Kotelawala Defence University (UHKDU) and the National Cancer Institute (NCI) for routine urine culture and antibiotic susceptibility testing (ABST) during the sample collection period. Among the 5270 urine samples received to UHKDU, 53 were CROs. Following the exclusion of three isolates due to mixed growth, 50 isolates from the UHKDU were included in the study. Further, a total of 2370 urine samples were received by NCI and 65 cultures were positive for CROs, of which 50/65 were included in our study following the exclusion of 15 isolates, due to the presence of mixed growth on the ABST plate and more than one urine sample received from the same patient. Therefore, a total of 100 CROs isolated from 100 patients with UTIs were included. Most of the isolates were from adult patients between the ages of 61 and 70 years (21.1%). While the mean age of the study sample was 52.6 ± 22.5 years, a male predominance (51.5%) was observed.

### 2.2. Organism Identification and Antibiotic Susceptibility Testing

**Figure 1:**
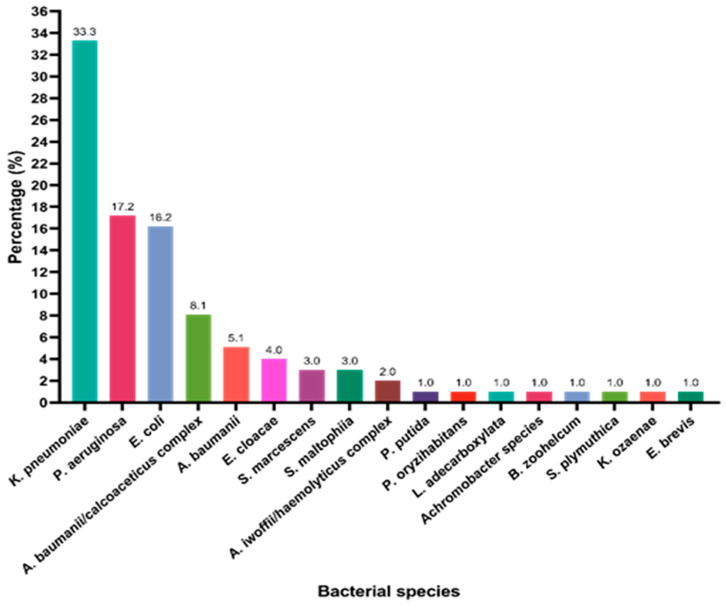
Distribution of different carbapenem-resistant organisms isolated from patients with urinary tract infections (*n* = 99). The full names of the bacterial species used include: *Klebsiella pneumoniae, Pseudomonas aeruginosa, Escherichia coli, Acinetobacter baumanii/calcoaceticus complex, Acinetobacter baumanii, Enterobacter cloaceae, Stenotrophomonas maltophilia, Acinetobacter iwoffii/haemolyticus, Pseudomonas putida, Pseudomonas oryzihabitans, Serratia marcescens, Leclercia adecarboxylata*, *Achromobacter* species, *Bergeyella zoohelcum, Serratia plymuthica, Empedobacter brevis, Klebsiella ozaenae*.

Among the 100 isolates, 99 were identified by the BD Phoenix automated system. The organism distribution among the study sample is shown in Figure 1. The majority were *Klebsiella pneumoniae*, accounting for 33.3% (*n* = 33/99) of the study sample, followed by *Pseudomonas aeruginosa* (17.2%, *n* = 17/99) and *Escherichia coli* (16.2%, *n* = 16/99).

### 2.3. Distribution of Carbapenemase-Encoding Genes in the Study Sample

**Figure 2:**
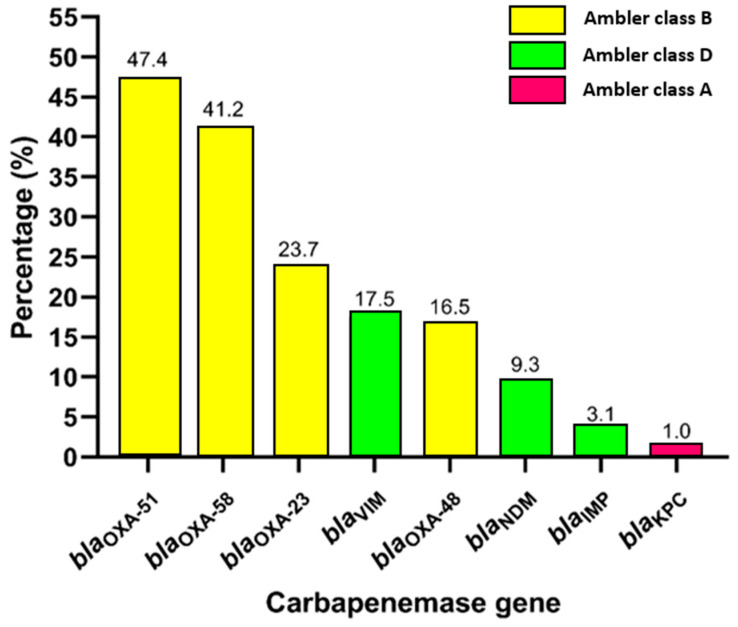
Overall distribution of Ambler class A, B and D carbapenemase genes among carbapenem-resistant uropathogens irrespective of their occurrence as single genes or combinations (*n* = 97).

Out of 99 isolates identified by the BD Phoenix automated system, a total of 97 isolates were randomly selected and carbapenemase genes were determined by a multiplex PCR kit. Therefore, two previously identified isolates (one; *P. aeruginosa* and one *E. coli*) were removed from the molecular analysis. The imipenem and meropenem resistance rates tested by the disk diffusion method prior to PCR testing (*n* = 97) showed 87.6% (*n* = 85/97) and 86.6% (*n* = 84/97), respectively, whereas the resistance rates obtained by the automated method were equal for both the carbapenem antibiotics (88.7%, *n* = 86/97). Among these 97 isolates, 74.2% (*n* = 72/97) were positive for the detected carbapenemase genes. A single gene was detected in 35.05% (*n* = 34/97), whereas two or more genes co-occurred in 39.18% (*n* = 38/97) of the isolates. Overall carbapenemase gene distribution, irrespective of its occurrence, as combinations or as a single gene, is shown in Figure 2. Accordingly, the majority of the isolated genes belonged to Ambler class D while the predominant gene was *bla*_OXA-51_ (47.4%, *n* = 46/97).

**Figure 3:**
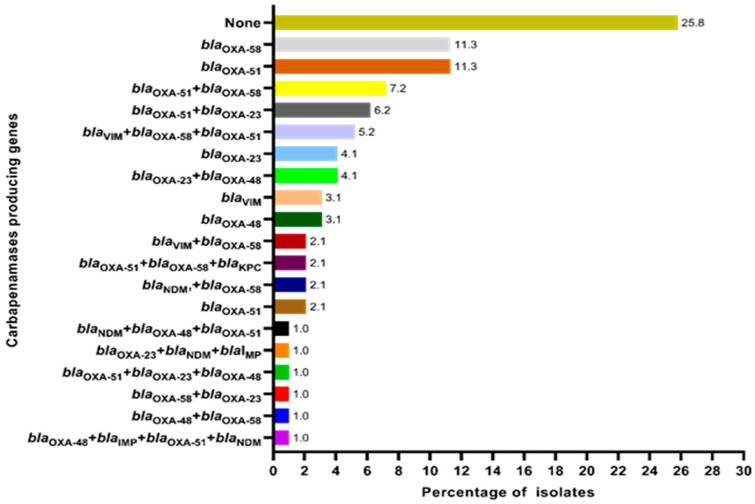
Percentage distribution of carbapenemase genes (as singles or combinations) identified among the included carbapenem-resistant uropathogens (*n* = 97).

The percentage distribution of carbapenemase genes among different uropathogens are presented in Figure 3. Among these genes, *bla*_OXA-51_ and *bla*_OXA-58_ showed the highest equal occurrence of 11.3% (*n* = 11/97). Genes such as; *bla*_VIM_, *bla*_OXA-23_, *bla*_OXA-48_, *bla*_OXA-51_, and *bla*_OXA-58_ were detected as single genes in different percentages. Two genes that commonly co-occurred were *bla*_OXA-51_ + *bla*_OXA-58_ (7.2%, *n* = 7/97). The predominant co-occurring gene combination among the three genes was *bla*_OXA-51_ + *bla*_OXA-58_ + *bla*_VIM_ (5.2%, *n* = 5/97). The maximum number of carbapenemase genes detected was four genes in the combination of *bla*_NDM_ + *bla*_IMP_ + *bla*_OXA-48_ + *bla*_OXA-51_ (1.0%, *n* = 1/97).

The most common genes detected in each CRO species irrespective of gene co-occurrences were *bla*_OXA-51_ (62.5%, *n* = 5/8) in *Acinetobacter baumannii*; *bla*_OXA-23_ and *bla*_OXA-51_ (60.0%, *n* = 3/5) in the *A. baumannii/calcoaceticus* complex; *bla*_OXA-51_ and *bla*_OXA-58_ (53.33%, *n* = 8/15) in *E. coli*; *bla*_OXA-51_ (35%, *n* = 7/20) in other GNB; *bla*_OXA-48_ (33.33%, *n* = 11/33) in *K. pneumoniae*; and *bla*_VIM_ (25.0%, *n* = 4/16) in *P. aeruginosa*. However, in the *A. baumannii/calcoaceticus* complex, *Achromobacter* spp., *Pseudomonas putida*, *Pseudomonas orizihabitans*, *Empedobacter brevis*, *Serratia plymuthica* and *Leclercia adecarboxylata*, all the carbapenemase genes were presented as gene combinations.

**Figure 4:**
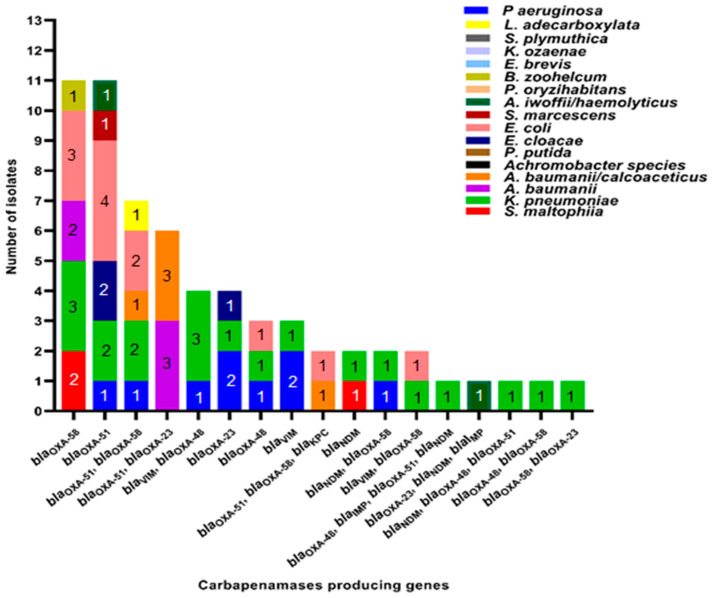
Carbapenemase gene distribution frequencies among the identified carbapenem-resistant uropathogens (each number in the bar graph represents the number of isolates among each category).

When gene combinations were considered, *bla*_OXA-51_ + *bla*_OXA-23_ was common in *A. baumannii* (60%, *n* = 3/5) and the *A. baumannii/calcoaceticus* complex (37.5%, *n* = 3/8). In *E. coli*, *bla*_OXA-51_ + *bla*_OXA-58_ was predominantly detected (13.3%, *n* = 2/15). Among *K. pneumoniae* strains, *bla*_VIM_ + *bla*_OXA-48_ was the most common gene combination (9.1%, *n* = 3/33). The highest gene diversity was observed in *K. pneumoniae*, which included 11 gene combinations. Four co-existing carbapenemase genes (*bla*_NDM_ + *bla*_IMP_ + *bla*_OXA-48_ + *bla*_OXA-51_, 3.0%) were also detected in an isolate of *K. pneumoniae*. It was isolated from a 73-year-old male patient admitted to the UHKDU. Figure 4 shows the frequencies of carbapenemase gene distribution among different carbapenem-resistant uropathogens.

### 2.4. Demographic Characteristics Versus Carbapenemase Gene Presence

When the carbapenemase gene distribution among different age groups was considered, *bla*_OXA-23_ was common among the 41–50 age group. The *bla*_OXA-58_-encoding genes were predominant among patients aged 51–60 years, whereas the *bla*_OXA-51_-encoding genes were predominant among patients aged 61–70 years and 71–80 years, as shown in Table 1. The most common carbapenemase genes among males and females were *bla*_OXA-58_ and *bla*_OXA-51_, respectively. The *bla*_KPC_ gene was not detected among females. A high frequency of coexisting genes was observed among males, as shown in Table 2. In terms of education level, the majority of the patients had a secondary education level (50/97, 51.54%) and were employed as skilled workers (46/97, 47.42%). The results of the univariate analysis are shown in Table 3. Only education level was significantly associated with the presence or absence of carbapenemase genes (*p* < 0.05), and it was subjected to binary logistic regression. Among the four education levels considered, education only up to primary education significantly contributed to carbapenemase gene presence (*p* < 0.05).

### 2.5. Clinical Characteristics Versus Carbapenemase Gene Presence

#### 2.5.1. Type of Current UTI

When considering the type of UTI, the majority of the patients (45/97, 46.4%) had UTIs attributed to other reasons such as catheter-associated UTIs, urosepsis, etc., followed by complicated pyelonephritis (30/97, 30.9%). In addition, out of the patients diagnosed with UTIs, the majority (72/97, 74.22%) were positive for one or more of the carabapenemase genes, without any statistically significant difference (*p* = 0.415) with the presence of carbapenemase genes, as shown in Table 2.

#### 2.5.2. Antibiotic Usage History

A total of 95.9% (*n* = 95/97) of patients were treated with outpatient antibiotic medications prior to hospital admission within the last 3 months for UTIs and other infections. However, only 38.8% (*n* = 34/95) were aware of the types of antibiotics used. The antibiotics used to treat these patients included co-amoxiclav, ceftriaxone, ciprofloxacin, clarithromycin, nitrofurantoin, metronidazole, gentamicin, and meropenem. However, the majority of the patients were treated with co-amoxiclav (47.1%, *n* = 16/34). Among the patients previously exposed to antibiotics, 73.7% were positive for carbapenemase genes. The only two patients who were not previously exposed to antibiotics were also positive for carbapenemase genes.

#### 2.5.3. History of Episodes of UTIs in the Past Six Months

Overall, 19.6% (19/97) of the patients had not experienced UTIs during the past six months. In addition, 11.3% (11/97) had experienced one UTI, and an equal number of patients (13.4%, *n* = 13/97) had previously experienced two or three episodes of UTIs. A total of 42.3% (*n* = 41/97) patients had experienced more than three previous episodes of UTIs during the past six months, and 68.3% (*n* = 28/41) were positive for carbapenemase genes. Furthermore, out of the 19.6% (*n* = 19/97) of patients who had not experienced previous UTI episodes, 78.9% *(n* = 15/19) were positive for at least one carbapenemase-encoding gene tested. However, a statistically significant difference was not noted (*p* = 0.593) between previous UTI episodes and the presence or absence of carbapenemase genes, as shown in Table 2.

#### 2.5.4. In-Hospital Clinical Improvement with the Presence of Carbapenemase Genes

Among the study sample, clinical improvement was observed in 23.7% (*n* = 23/97) patients by day three. Of these improved patients, 30.4% (*n* = 7/23) harbored a single carbapenemase-encoding gene, while 47.8% (*n* = 11/23) carried multiple carbapenemase-encoding genes. By day five of treatment, 58.1% (*n* = 43/74) of the patients demonstrated clinical improvement, and an equal proportion were (41.9%, *n* = 18/43) harboring either single or multiple carbapenemase genes. At day seven, post-treatment with a sensitive antibiotic, 90.3% (*n* = 28/31) of patients exhibited clinical improvement. Similar to the day five findings, an equal distribution of patients harbored single or multiple carbapenemase genes (32.1%, *n* = 9/28). Accordingly, a progressive increase in clinical improvement was observed during the period of hospital stay. However, three patients were observed to have worsening clinical condition following day seven of treatment, and all of them were found to carry multiple carbapenemase-encoding genes. Moreover, a statistically significant difference was not noted (*p* > 0.05) between the in-hospital clinical improvement and the presence of carbapenemase genes, as shown in Table 2.

#### 2.5.5. Clinical Improvement Following Hospital Discharge with the Presence of Carbapenemase Genes

Overall, 71.1% (*n* = 69/97) of the patients experienced UTI symptoms following hospital discharge, of whom 52.2% (*n* = 36/69) were managed with outpatient department (OPD) medications, and an equal proportion (39.1%, *n* = 27/69) harbored carbapenemase-encoding genes. Additionally, 50.5% (*n* = 49/97) of patients required re-admission to general wards with no intensive care unit (ICU) admissions recorded. Among the re-admitted patients, 30.6% (*n* = 15/49) harbored single carbapenemase-encoding genes, while 44.9% (*n* = 22/49) harbored multiple genes. The majority (93.9%, *n* = 46/49) were discharged following complete recovery. However, 6.1% (*n* = 3/49) developed infectious complications, including one patient without detectable carbapenemase genes and two patients with multiple carbapenemase-encoding genes. Furthermore, none of the considered clinical improvement-related factors were significantly associated with the presence of carbapenemase genes (*p* > 0.05), as shown in Table 2.

#### 2.5.6. Deaths Associated with the Presence of Carbapenemase Genes

Overall, 10.3% (n = 10/97) of the patients died during the study period, although mortality was attributed to non-UTI related causes. As summarized in Table 3, the majority of the deceased patients were females (60.0%, *n* = 6/10) and of advanced age. The most frequently observed CRO was *K. pneumoniae*. Notably, three of the deceased patients who were deceased did not harbor any carbapenemase-encoding genes as shown in Table 3.

## 3. Discussion

UTIs represent a significant health concern worldwide with increasing resistance to common antibiotics, creating a substantial challenge in the management of patients [23,24]. Primarily used antibiotics to treat UTIs are nitrofurantoin, amoxicillin, trimethoprim/sulfamethoxazole, and ciprofloxacin. However, due to the increasing prevalence of antibiotic resistance among uropathogens, carbapenems have been employed as an alternative therapeutic option. Recently, the emergence of CROs in UTIs has been reported, posing a significant clinical challenge. Among the various resistance mechanisms, the production of carbapenemases is a critical concern due to their ability to hydrolyze a broad spectrum of β-lactam antibiotics, including carbapenems [25]. The global trends in CROs show an alarming increase across multiple pathogens and regions [26]. In the present study, we assessed molecular determinants of urinary CROs and their clinical characteristics. These isolates were recovered from hospitalized patients in two Sri Lankan hospitals, the UHKDU and the NCI, from January to December 2023. Among the 7640 urine samples screened, 100 CROs were included in this study based on the inclusion and exclusion criteria, of which 99/100 were identified to the species level via the BD Phoenix automated system. The genotypic analysis by a multiplex PCR kit was performed for 97/99 isolates and 72/97 (74.2%) were identified as carbapenemase producers.

In the present study, a high occurrence of CROs was observed among males, supporting previous findings [20,27]. Although UTIs are more common among females [1], the reason for this high CRO occurrence among males may be that older males have an increased risk of experiencing prostate-related infections such as benign prostatic hyperplasia (BPH), which may cause urine incontinence and recurrent UTIs. In addition, due to prostate enlargement, urine catheterization is needed. As a result, repeated and chronic antibiotic treatments are needed, resulting in resistance. In addition, the highest occurrence of CROs was observed among elderly patients, supporting previous findings [28]. This may be due to a weakened immune system, recent hospitalizations, comorbidities, chronic diseases, and frequent antibiotic usage with increasing age. In contrast, a higher carbapenemase production rate was reported among children aged less than one year in neonatal and pediatric wards [27].

Carbapenem resistance is particularly concerning in GNB such as *K. pneumoniae*, *P. aeruginosa* and *A. baumannii* [29]. Our study detected a wide diversity of carbapenemase producers, with *K. pneumoniae* being the most common uropathogen (33.2%), which resonates with studies previously published in Sri Lanka [20,21,22] and Sudan [27]. In contrast, different findings were also reported in Germany [30]. *K. pneumoniae* was followed by *P. aeruginosa*, *E. coli*, the *A. baumannii/calcoaceticus* complex, *A. baumannii,* and some other GNB, which is in line with previous findings [26,30]. *A. baumannii* revealed high crabapenem resistance rates globally with an upward trend in 2020 to 2023 supporting our findings [31]. In addition, the isolation of some less frequent carbapenemase producers in our study is important, primarily due to their potential to disseminate carbapenemase genes and act as reservoirs for carbapenemases.

Genotypic analysis by PCR revealed that 74.2% (*n* = 72/97) of the tested uropathogenic CROs were positive for carbapenemase-encoding genes, which is higher than the rate reported in Sudan (58.7%) [27] and Uganda (24.4%) [32]. The tested eight carbapenemase-encoding genes, i.e., *bla*_NDM_, *bla*_KPC_, *bla*_IMP_, *bla*_VIM_, *bla*_OXA-51_, *bla*_OXA-23_, *bla*_OXA-48_ and *bla*_OXA-58_, presented as single genes and in different combinations, indicating high carbapenem resistance variability in our study sample. In addition, the present study reported a higher occurrence rate of gene combinations (39.18%) than single genes, supporting the findings of Egypt, which reported a higher substantial gene co-occurrence rate of 54.5% than that of single-gene detections (45.5%) [31]. The reason for this may be that these genes are carried on mobile genetic elements and are transmissible easily within different hospital settings. In addition, the diverse nature of *bla*_NDM-1_ and its capability to be carried on plasmids make it a potential source of MDR [31]. Furthermore, 25.77% (*n* = 25/97) of carbapenem-resistant isolates detected by disk diffusion were PCR-negative for carbapenemase genes. This can be attributed to non-carbapenemase-mediated resistance mechanisms such as porin loss, over-expression of efflux pumps, and alteration in penicillin-binding proteins [33,34]. Additionally, the mere presence of resistance genes does not confer phenotypic resistance. Gene expression can vary depending on regulatory mechanisms, bacterial physiology and environmental conditions. Therefore, the integration of phenotypic tests with genotypic methods is important to fully characterize resistance profiles.

Different carbapenemase-producing genes, such as *bla*_OXA-181_ [14,17], *bla*_OXA-48-like_ [20] and *bla*_NDM_ [21], have been reported in Sri Lanka, whilst *bla*_NDM_, *bla*_OXA-48_, *bla*_KPC_, *bla*_VIM_, *bla*_OXA-51_, *bla*_OXA-58_ were reported in Sudan [27], Egypt [31], Uganda [32], and South India [35] in different percentages. In addition, our study detected one *E. coli* isolate harboring *bla*_KPC_ (1.0%) as a combination of *bla*_KPC_ + *bla*_OXA-51_ + *bla*_OXA-58_, and it was isolated from a 70-year-old male patient, supporting the available local data [20,21,22]. Similarly, the low occurrence of *bla*_KPC_ was discussed in Anbazhagan et al. (2024) [35]. In the present study, *bla*_NDM_ did not occur as a single gene, but four combinations were noted. Higher *bla*_NDM_ occurrence percentages than our study were reported locally [20] and internationally [36]. Moreover, *bla*_NDM-1_ and *bla*_NDM-4-like_ variants have also been reported in Sri Lanka, India, and Pakistan [10,14]. Although previously only two combinations of *bla*_NDM_, such as *bla*_NDM_ + *bla*_OXA-48_ and *bla*_NDM_ + *bla*_KPC_ + *bla*_OXA-48_, were reported in Sri Lanka, the present study reported four co-occurrences of *bla*_NDM_ with *bla*_OXA-48_, *bla*_OXA-23_, *bla*_OXA-51_ and *bla*_IMP_ in different combinations, supporting the findings of other countries [27,37,38]. Similar percentages of *bla*_IMP_, *bla*_VIM_, *bla*_OXA-23_, *bla*_OXA-51_ and *bla*_OXA-58_ were also reported in studies published in China [37], Thailand [39], Pakistan [40], etc. Although *bla*_IMP_ occurrence in our study was 17.5%, a higher rate (32.4%) was reported by Tuhamize et al. (2024) [32], making it the most prevalent gene in their study. Interestingly, the *bla*_OXA-23_ + *bla*_OXA-51_ combination was predominant among the *A. baumanni* (60%) and *A. baumannii/calcoaceticus* complex isolates (37.5%). Similarly, high percentages were detected in Pakistan and China, suggesting *bla*_OXA-23_ and *bla*_OXA-51_ as the major cause of carbapenem susceptibility in *A. baumanni* [41,42]. To date, local studies reporting the occurrence of these *bla*_IMP_, *bla*_VIM_, *bla*_OXA-23_, *bla*_OXA-51_, and *bla*_OXA-58_ genes are scarce. This gap in data may be due to the lack of prior screening of carbapenemase-encoding genes in the Sri Lankan clinical setting.

Univariate analysis revealed education level as the only factor significantly associated with the presence of carbapenemase genes. Further analysis using binary logistic regression confirmed that patients with education only up to the primary level were at significantly high risk of acquiring uropathogens with carbapenemase-encoding genes. This finding aligns with the available evidence suggesting that lower educational attainment correlates with limited awareness of hygiene and infection prevention practices and delayed healthcare-seeking behavior, which may lead to increased susceptibility to MDR infections [43,44]. However, education level is a broad aspect, which may reflect health literacy or access to care, but it does not directly measure antibiotic-related behaviors such as self-medication, adherence, or awareness, which are more directly linked to antimicrobial resistance. Notably, the small sample sizes in each education subgroup, in particular the groups with no schooling (*n* = 3), only primary education (*n* = 23), and higher education (*n* = 23), limit the strength of the conclusions. While the patients with higher education might be expected to demonstrate better antibiotic-related practices due to greater health awareness, the study lacked sufficient statistical power to detect it. Although published studies reported a correlation between lower education with poor knowledge and inappropriate usage of antibiotics [40,41], most were focused on behavioral outcomes rather than the presence of resistance genes. Therefore, this noteworthy association should be interpreted cautiously, considering broader social and behavioral determinants of antibiotic resistance.

Significant associations were not observed between the presence of carbapenemase genes and other clinical or demographic factors such as age, gender, occupation or type of UTI. Although age and gender were commonly considered as risk factors in published infectious disease studies, their influence on the carriage of carbapenemase genes remains inconsistent globally. Higher antibiotic resistance rates were reported in the elderly population due to frequent hospitalizations, longer hospital stays, and antibiotic exposure [45,46,47], whilst UTIs are generally prevalent among females mainly due to anatomical predisposition [1]. Antibiotic-resistant patterns, particularly carbapenem resistance, are more likely to be influenced by other factors such as length and frequency of hospitalization, previous antibiotic use, and underlying co-morbidities such as diabetes, chronic kidney disease than by gender alone [46]. Additionally, although ethnicity was not considered in our study, its role in the frequency of carbapeneamase-encoding bacteria has been reported due to the variation in microbial species colonizing patients from different geographical regions [45].

Previous episodes of UTIs and prior antibiotic treatments were not significantly associated with the presence of carbapenemase genes in our study, although the history of antibiotic use may contribute to the emergence of carbapenem resistance [48,49]. However, the lack of statistically significant difference in our study could be due to diversified reasons such as unawareness of patients about the medicines they are taking, limited sample size, and inclusion of immunocompromised patients with malignancies. In addition, the occurrence of potential carbapenem resistance in Sri Lanka was facilitated by inappropriate antibiotic prescriptions (22.6%) and redundant antibiotic therapy (17.1%) against local treatment guidelines [50]. This ultimately leads to high mortality and morbidity rates due to treatment failure outbreaks of antibiotic-resistant infections, and increased healthcare-associated costs [12]. In terms of clinical characteristics, neither clinical improvement during hospital stay nor clinical improvement following hospital discharge was significantly associated with the presence of carbapenemase genes.

Among the 97 enrolled patients, mortality was observed in 10 cases (10.3%), with four deaths occurring during hospitalization and six following hospital discharge. Out of the post-discharge deaths, four occurred within 30 days, while the remaining two deaths were recorded within 30–60 days and 60–90 days post-discharge, respectively, although, none of these fatalities were attributed to UTIs. Demographic analysis revealed that mortality was more prevalent among female patients and older individuals. Notably, three deceased patients showed no detectable carbapenemase genes. These findings are in contrast with the published literature demonstrating higher mortality rates due to infections among patients with CROs compared to those infected with carbapenem-susceptible organisms [51]. Additionally, the clinical outcome may depend on the type of carbapenemase genes present. In particular, isolates encoding *bla*_NDM-1_ or *bla*_NDM-1_/*bla*_OXA-48_ presented higher mortality rates than *bla*_OXA-48_ producers did. Since these genes confer high MICs with high levels of resistance to carbapenems and other β-lactam antibiotics, delays may occur in diagnosis and initiation of effective therapy leading to poor outcomes and increased mortality. In addition, the dissemination of plasmids carrying these genes makes pathogens refractory to the commonly used antibiotics [52,53]. Notably, our findings demonstrate that the mere presence of carbapenemase genes does not independently predict mortality outcomes. This suggests that infections caused by CROs may contribute to overall clinical deterioration, complicating the management of comorbid conditions and leading to adverse outcomes. Furthermore, we attempted to correlate the presence of carbapenem resistance genes with mortality, recognizing that the genetic determinants of resistance could play a role in the severity and treatment challenges of the infections, potentially influencing patient survival indirectly. Our findings demonstrate that the mere presence of carbapenemase genes does not independently predict mortality outcomes. The treatment of CRO-associated UTIs should be decided based on the presence or absence of carbapenemase genes, their mechanisms of resistance, and careful clinical monitoring of patient response, especially when using carbapenems or colistin as therapeutic drugs. However, no statistically significant difference was documented in the mortality outcome of patients infected with CROs when treated with different antimicrobial agents, supporting our findings [52,54].

The present study reports the detection of eight carbapenemase-encoding genes via a multiplex PCR kit to detect urinary CROs in Sri Lanka. This is the major strength of our study, which enabled the early identification of causative genetic mechanisms, early initiation of appropriate antibiotic therapy, and preventive measures. Moreover, mortality associated with inappropriate, prolonged antibiotic prescriptions is limited by the use of multiplex PCR systems [55,56].

Despite these strengths, there were several limitations as well. The study sample size is a major limitation. Although this study included 100 patients from two hospitals, the BD Phoenix automated identification was performed for 99 isolates while PCR testing was performed for only 97 isolates due to budgetary limitations. Further studies need to be conducted, including large sample sizes, in different geographical regions, with resource-heavy settings. To avoid the limitation that occurred due to exclusion of samples with mixed growth, those isolates should be purified to obtain a pure culture. In addition, the broader epidemiology of CROs in the country may be identified by testing other clinical samples as well. Here, we tested only carbapenemase production, considering eight genes. The other known carbapenemase genes were not tested considering their low prevalence, limited geographic distribution and minimal detection in clinical uropathogenic isolates, particularly in South Asian settings. The particular PCR kit (Hi-PCR carbapenemase gene probe multiplex PCR kit, catalog no: MBPCR132) was selected as it was designed in India which has similar antibiotic resistance patterns and is geographically similar and the closest country to Sri Lanka. Despite the high prevalence of *bla*_OXA-181_ in this region, its exclusion underestimates the true burden of carbapenem resistance in the country. Therefore, further studies should be conducted by incorporating these targets in future surveillance efforts and whole-genome sequencing as a comprehensive alternative. Moreover, the detection of other antimicrobial resistance mechanisms such as efflux pumps, mutations in porins and penicillin-binding proteins, at least in PCR-negative isolates, may contribute to the overall resistance profile. While the molecular detection by PCR is valuable in identifying the presence of resistance genes, they do not confirm whether these genes are actively expressed or confer functional resistance. Thus, further studies related to protein-level detection methods, such as enzyme activity assays and immunoassays, are currently underway by the same research team to confirm functional resistance. These findings would enhance the clinical relevance and diagnostic accuracy of molecular findings [57]. At the time of treatment, the carbapenem-resistant gene composition data were not available. However, the presence/absence of co-morbidities were taken into consideration by clinicians when making treatment decisions. Therefore, we were unable to determine the impact of genetic composition on the treatment. Further studies could be encouraged to detect the association of the number of carbapenem-resistant genes present with the resistance level exhibited by the respective CRO, the variation in treatment strategy according to number and type of genes present and comorbidities.

The emergence and dissemination of UTIs caused by CROs possess a significant public health threat, particularly in low- and middle-income countries such as Sri Lanka. Since these infections are difficult to treat, due to limited availability of therapeutic options, they often demand the use of last-resort antibiotics, which may be unavailable or unaffordable in resource-limited settings. Additionally, Sri Lanka is a best-fitting and well-known country for the tourism industry and it plays a significant role in Sri Lanka’s economy. As a result of this high influx of foreign travelers from countries where carbapenemase-encoding genes are endemic such as India, there is an increased potential for the dissemination of these resistance genes, which poses a risk not only to locals [34] but also to global health [51]. Furthermore, the silent community transmission of CROs contributes to the undetected spread of carbapenemase genes, which may further complicate infection control strategies. As a result, increased mortality and morbidity, prolonged hospital stays and elevation of healthcare-associated costs may occur globally. Evidence-based public health policies, proper antibiotic stewardship programs, robust surveillance, and timely interventions play crucial roles in combating the threat of carbapenem resistance. Knowledge of resistance mechanisms is a cornerstone for reducing carbapenem resistance. Alternative β-lactamase inhibitor drug combinations such as ceftazidime/avibactam, which are highly effective against *bla*_OXA-48_, and the addition of aztreonam are needed to treat *bla*_NDM-1_ [25]. In addition, the reuse of older forgotten antibiotics and the development of new antibiotics would be beneficial in both local and global contexts.

## 4. Materials and Methods

A hospital-based prospective, cross-sectional study was conducted in two hospitals in the Western province of Sri Lanka; UHKDU and NCI. The study period was from January to December 2023. The urine culture samples received by the respective microbiology laboratories were scrutinized daily as a part of routine diagnosis. The antibiotic susceptibility pattern of each isolated uropathogen was detected, following standard operating procedures by the laboratory staff. The study investigators selected urinary isolates from UTI patients that showed ≥10^4^ colony-forming units (CFU)/mL and demonstrated resistance and/or intermediate resistance to meropenem and/or imipenem according to the CLSI disk diffusion method. Therefore, urine samples were not collected and processed specifically for the current research. Patients who were not presented with UTIs, isolates with mixed growth in urine cultures, and repeated isolates from the same patient were excluded. Following the isolation of urinary CROs, further laboratory analysis and clinical characteristics assessment were performed.

### 4.1. Screening for Carbapenem Resistance in Uropathogens by the Disk Diffusion Test

Zone diameters of meropenem and imipenem were interpreted according to 2023, CLSI guidelines [58]. Organisms that showed resistance or intermediate resistance to imipenem and/or meropenem antibiotic disks on Mueller‒Hinton agar plates were considered as possible CROs. Antimicrobial susceptibility testing was quality controlled with the *E. coli* ATCC 25922 strain during the screening period, according to CLSI recommendations.

### 4.2. Organism Identification and Susceptibility Testing by the BD Phoenix Automated System

The BD Phoenix^TM^ NMIC/ID-421, MD, USA panel, which is specialized for the detection of carbapenemase-producing organisms, was used for urinary CRO identification and susceptibility testing of imipenem and meropenem in the BD Phoenix automated system. Tests were performed following the manufacturers’ instructions, and the quality control was performed using the *E. coli* ATCC25922 strain.

### 4.3. Bacterial DNA Extraction

Bacterial genomic DNA was extracted using a column-based purification method. We used the HiPuraA^®^ bacterial genomic extraction kit (catalog no: MB505), manufactured by Himeia Laboratories in India. Briefly, carbapenem-resistant isolates were sub-cultured on blood agar plates and suspended in 5% peptone broth the following day. Bacterial cells were palleted by centrifugation at 13,000 rpm for 2 min and the supernatant was discarded. The pallet was resuspended in lysis buffer and treated with 20 µL of Proteinase K solution, followed by incubation at 55 °C for 30 min. RNase A treatment was performed by adding 20 µL of RNase A solution and incubated for 5 min at room temperature. Following lysis of cells, ethanol was added to the lysate and centrifugated. Then, the lysate was loaded to the spin column (HiElute Miniprep spin column). Following centrifugation, pre-washing and washing steps were performed using the respective buffers. After drying columns by centrifugation, DNA was eluted using elution buffer and stored at −20 °C. Prior to PCR testing, DNA quantification was performed.

### 4.4. Detection of Carbapenem-Resistant Genes

The extracted DNA was analyzed by PCR using the Hi-PCR carbapenemase gene probe multiplex PCR kit (catalog no: MBPCR132) manufactured by Himedia laboratories in India. A total of eight genes conferring resistance to carbapenems (*bla*_NDM_, *bla*_KPC_, *bla*_IMP_, *bla*_VIM_, and *bla*_OXA-48_) were analyzed, since they are the most clinically reported and widespread carbapenemase genes across the globe, particularly in South and Southeast Asia, whilst *bla*_OXA-23_, *bla*_OXA-51_, and *bla*_OXA-58_ were tested as they are commonly detected in *A. baumannii*, especially in South Asia. Also, *A. baumannii* is a WHO priority pathogen epidemiologically linked with healthcare-associated outbreaks and limited evidence is available on its occurrence in Sri Lanka.

The bacterial DNA samples were tested in two PCR tubes, namely carbapenem-resistant gene-1 (CRG1) and CRG2, each targeting four genes. Master mixes for CRG1 and CRG2 were prepared in PCR tubes according to manufacturer’s instructions, each containing 12.5 µL of Hi-Quanti 2X real-time PCR master mix, 4 µL of either CRG1 and CRG2 primer-probe mix, 1 µL of internal control DNA, 1.5 µL of molecular biology grade water, and 5 µL of template DNA. The final reaction volume per tube was 25 µL. The PCR tubes were carefully labeled, and the particular reaction mixtures were added and loaded into the Bio-Rad CFX Maestro real-time PCR machine for amplification. Each PCR run was quality controlled using positive and negative controls, which were provided with the PCR kit.

### 4.5. Demographics, Clinical Factors and Clinical Outcome Assessment

A recently validated questionnaire by the same authors was used to collect demographic and clinical data from the selected group of patients, and the questionnaire was completed. Here, higher education was defined as the attainment of any degree or diploma-level qualification following completion of secondary education. Clinical outcome assessment during the hospital stay was assessed by visiting wards at three, five, and seven days of intervals; subsequently, urine culture reports confirmed the presence of CROs. The parameters considered for in-ward clinical improvement assessment were: the patients’ clinical response as indicated in the patients’ records, reduction in white blood cell (WBC) count, and C-reactive protein level. Data on these parameters were obtained by referring to patients’ medical records, inputs from treating medical officers, and laboratory reports.

The clinical outcomes of patients following hospital discharge were collected by contacting patients/their guardians over the phone. Written informed consent to participate in the study and contact over the phone were obtained from patients/their guardians at the first ward visit. They were followed for up to three months and were contacted at days 30, 60 and 90 following hospital discharge. The patients/their guardians were questioned regarding the recurrence of UTI symptoms such as dysuria, increased urinary frequency, abdominal pain, fever, back pain, loin pain, nausea, vomiting, and confusion during this period. In addition, questions related to the type of medical treatment received for recurrent UTIs, such as outpatient department (OPD) treatment, ward treatment due to UTI recurrence, and mortality data during the 3 months, were also obtained.

### 4.6. Statistical Analysis

Data analysis was performed via IBM SPSS software version 26, Armonk, NY, USA. The means and standard deviations were used to summarize age. Frequencies and percentages are reported for categorical variables. The correlations between ordinal and non-normal continuous variables were analyzed by the Spearman correlation coefficient, whereas Pearson’s chi-square test (or Fisher’s exact test for variables with <5 sample counts) was used to analyze the significance of categorical data. Associations of different genetic determinants with patient demographics and clinical outcomes were analyzed by dividing the study sample into two groups: presence or absence of carbapenem resistance genes. Statistically significant demographic/clinical variables, with the presence or absence of carbapenem resistance genes identified by univariate logistic regression, were further subjected to binary logistic regression. A significance value (*p*) < 0.05 was considered significant.

## 5. Conclusions

This study highlights the presence of diverse carbapenemases in UTI patients and their clinical characteristics in Sri Lanka. Here, we detected a carbapenemase gene positivity rate of 74.2% with *bla*_OXA-51_ (47.4%) being the predominant gene occurrence. *K. pneumoniae* (33.2%) was the most common CRO. The primary education attainment was statistically detected as a predictor of carbapenemase gene presence. To the best of our knowledge, this the first study in Sri Lanka which assessed eight carbapenemases genes simultaneously among UTI patients. Understanding the local and global epidemiology and resistance mechanisms of CROs is important in therapeutic approaches. The detection of carbapenemase-encoding genes as singles and co-occurrences emphasizes the complex nature of genetic composition which might be helpful in treating UTIs caused by CROs. Consideration of mortality rate and determination of causative factors are of utmost important in improving patient care. Findings emphasize the need for continuous surveillance, infection control measures, and antibiotic stewardship programs to minimize further transmission and future outbreaks of urinary CROs in clinical settings.

## Figures and Tables

**Table 1:** Distribution of carbapenemase genes among different age categories and genders within the study sample (*n* = 97).

Carbapenemases Encoded by Gene/s	Age Categories in Years										Gender	
	0–10	11–20	21–30	31–40	41–50	51–60	61–70	71–80	81–90	91–100	Males	Females
*bla*_NDM_	1	0	0	0	0	1	0	0	0	0	0	2
*bla*_VIM_	0	0	1	1	1	0	0	0	0	0	2	1
*bla*_OXA-51_	0	0	2	0	1	2	2	3	1	0	3	8
*bla*_OXA-23_	0	1	0	1	0	1	1	0	0	0	1	3
*bla*_OXA-48_	0	0	0	0	0	1	2	0	0	0	1	2
*bla*_OXA-58_	1	1	0	1	0	5	1	1	0	1	6	5
*bla*_NDM_, *bla*_OXA-58_	0	0	0	1	0	1	0	0	0	0	1	1
*bla*_VIM_, *bla*_OXA-48_	1	0	1	0	0	0	0	2	0	0	4	0
*bla*_VIM_, *bla*_OXA-58_	1	0	0	0	0	0	0	1	0	0	1	1
*bla*_OXA-51_, *bla*_OXA-23_	1	0	0	1	2	1	1	0	0	0	2	4
*bla*_OXA-51_, *bla*_OXA-58_	1	1	1	0	0	0	2	2	0	0	4	3
*bla*_OXA-48_, *bla*_OXA-58_	0	0	0	0	0	0	0	1	0	0	1	0
*bla*_OXA-48_, *bla*_OXA-23_	0	0	0	0	2	1	0	1	0	0	2	2
*bla*_OXA-58_, *bla*_OXA-23_	0	0	0	0	0	0	0	1	0	0	1	0
*bla*_OXA-51_, *bla*_OXA-23_, *bla*_OXA-48_	0	0	0	0	0	0	0	1	0	0	1	0
*bla*_VIM_, *bla*_OXA-58_, *bla*_OXA-51_	0	0	0	1	0	0	2	1	1	0	3	2
*bla*_OXA-51_, *bla*_OXA-58_, *bla*_KPC_	0	0	0	0	0	0	1	0	1	0	1	0
*bla*_OXA-23_, *bla*_NDM_, *bla*_IMP_	0	0	0	1	0	0	0	0	0	0	1	0
*bla*_NDM_, *bla*_OXA-48_, *bla*_OXA-51_	0	0	0	0	0	0	1	0	0	0	1	0
*bla*_OXA-48_, *bla*_OXA-51_, *bla*_IMP_, *bla*_NDM_	0	0	0	0	0	0	0	1	0	0	1	0
No carbapenemase-encoding genes detected	1	1	1	1	5	4	8	4	0	0	12	13
Total	7	4	6	8	11	17	21	19	3	1	50	47

**Table 2:** Univariate analysis of demographic- and clinical outcome-related factors with the presence or absence of carbapenemase genes among patients with urinary tract infections caused by carbapenem-resistant organisms.

Variables, *n* (%)				Total (*n* = 97)	CRG Present (*n* = 72, %)	CRG Absent (*n* = 25, %)	*p* Value
Demographic factors (*n* = 97)	Age (mean ± SD, years = 52.6 ± 22.5)	0–10		7	6 (85.7%)	1 (14.3%)	0.634
		11–20		4	3 (75.0%)	1 (25.0%)	
		21–30		6	5 (83.3%)	1 (16.7%)	
		31–40		8	7 (97.5%)	1 (12.5%)	
		41–50		11	6 (54.5%)	5 (45.5%)	
		51–60		17	13 (76.5%)	4 (23.5%)	
		61–70		21	13 (61.9%)	8 (38.1%)	
		71–80		19	15 (78.94%)	4 (21.1%)	
		81–90		3	3 (100%)	0	
		91–100		1	1 (100%)	0	
	Gender	Male		50	38 (76.0%)	12 (24.0%)	0.680
		Female		47	34 (72.3%)	13 (27.7%)	
	Education level	Primary		23	19 (82.6%)	4 (17.4%)	0.034 *
		Secondary		50	31 (62.0%)	19 (38.0%)	
		Higher education		21	19 (90.5%)	2 99.5%)	
		No schooling		3	3 (100%)	0	
	Occupation	Unemployed		23	18 (78.26%)	5 (21.74%)	0.680
		Retired		4	2 (50.0%)	2 (50.0%)	
		Unskilled worker		10	7 (70.0%)	3 (30.0%)	
		Skilled worker		46	34 (73.9%)	12 (23.1%)	
		Professional or managerial		7	5 (71.4%)	2 (28.6%)	
		Not applicable		7	6 (85.71%)	1 (14.29%)	
Clinical presentation and history-related factors (*n* = 97)	Type of current urinary tract infection	Uncomplicated cystitis		4	4 (100%)	0 (0%)	0.415
		Complicated cystitis		16	13 (81.3%)	3 (18.8%)	
		Uncomplicated pyelonephritis		2	2 (100%)	0	
		Complicated pyelonephritis		30	23 (76.7%)	7 (23.3%)	
		Other		45	30 (66.7%)	15 (33.3%)	
	Outpatient antibiotic treatment prior to admission for the current illness	Yes		95	70 (73.7%)	25 (26.3%)	0.400
		No		2	2 (100%)	0	
	History of episodes of UTI in last 6 months	None		19	15 (78.9%)	4 (21.1%)	0.593
		One		11	10 (90.9%)	1 (9.1%)	
		Two		13	10 (76.9%)	3 (23.1%)	
		Three		13	9 (69.2%)	4 (30.7%)	
		More than three		41	28 (68.3%)	13 (31.7%)	
Clinical outcome assessment during hospital stay (n = 97)							
Clinical improvement during hospital stay	Clinical improvement at day 3 of treatment	Yes		23	18 (78.3%)	5 (21.7%)	0.613
		No		74	54 (72.9%)	20 (27.0%)	
	Clinical improvement on day 5 treatment	Yes		43	34 (79.1%)	9 (20.9%)	0.459
		No		31	22 (70.9%)	11 (35.5%)	
	Clinical improvement at day 7 of treatment	Yes		28	18 (64.3%)	10 (35.7%)	0.251
		No		3	3 (50.0%)	0	
	Worsening after day 7 of treatment	Yes		3	3 (100%)	0	0.402
		No		90	68 (75.6%)	22 (24.4%)	
Death	Death during hospital stay	Yes	Due to UTI	0	0	0	0.971
			Due to other reasons	4	3 (75.0%)	1 (25.0 0%)	
		No		93	69 (74.2%)	24 (25.8%)	
Clinical outcome assessment following hospital discharge (n = 93)							
UTI symptom recurrence following hospital discharge	UTI symptom recurrence within 30 days of infection	Yes		33	25 (75.8%)	8 (24.2%)	0.761
		No		60	47 (78.3%)	13 (21.7%)	
	UTI symptom recurrence within 30–60 days of infection	Yes		24	21 (87.5%)	3 (12.5%)	0.288
		No		69	51 (73.9%)	18 (26.1%)	
	UTI symptom recurrence within 60–90 days of infection	Yes		12	8 (66.7%)	4 (33.3%)	0.260
		No		81	64 (79.0%)	17 (20.9%)	
OPD treatment for UTI following hospital discharge	OPD treatment for UTI within 30 days of infection	Yes		19	15 (78.9%)	4 (21.1%)	0.591
		No		74	57 (77.0%)	17 (22.9%)	
	OPD treatment for UTI with 30–60 days	Yes		11	8 (72.2%)	3 (27.3%)	0.904
		No		82	64 (78.1%)	18 (21.9%)	
	OPD treatment within 60–90 days for UTI infection	Yes		6	3 (50.0%)	3 (50.0%)	0.161
		No		87	69 (79.3%)	18 (20.7%)	
Re-admissions due to UTI during three months	Hospital admission due to UTIs within 30 days of infection	Yes	Ward admission	26	19 (73.1%)	7 (26.9%)	0.699
			ICU admission	0	0	0	
		No		67	53 (79.1%)	14 (20.9%)	
	Hospital admission due to UTIs within 30–60 days of infection	Yes	Ward admission	17	15 (88.2%)	2 (11.8%)	0.223
			ICU admission	0	0	0	
		No		76	57 (75.0%)	19 (25.0%)	
	Hospital admission due to UTIs within 60–90 days of infection	Yes	Ward admission	6	3 (50.0%)	3 (50.0%)	0.171
			ICU admission	0	0	0	
		No		87	69 (79.3%)	18 (20.7%)	
Outcome if re-admitted due to UTI during three months	If admitted outcome within 30 days	Complete recovery		24	18 (75.0%)	6 (25.0%)	0.310
		Complicated infection		2	0	2 (100%)	
		No hospital admission		67	53 (79.1%)	14 (20.9%)	
	If admitted outcome within 30 to 60 days of infection	Complete recovery		16	14 (87.5%)	2 (12.5%)	0.525
		Complicated infection		1	1 (100%)	0	
		No hospital admission		76	57 (75.0%)	19 (25.0%)	
	If admitted outcome within 60–90 days of infection	Complete recovery		6	4 (66. 7%)	2 (33.3%)	0.455
		Complicated infection		0	0	0	
		No hospital admission		87	72 (82.8%)	15 (17.2%)	
Deaths occurred during three months	Death within 30 days	Yes	Due to UTI	0	0	0	0.265
			Due to other reasons	4	2 (50.0%)	2 (50.0%)	
		No		93	69 (74.2%)	24 (25.8%)	
	Death within 30–60 days	Yes	Due to UTI	0	0	0	0.554
			Due to other reasons	1	1 (100%)	0	
		No		96	71 (74.0%)	25 (26.0%)	
	Death within 60–90 days	Yes	Due to UTI	0	0	0	0.554
			Due to other reasons	1	1 (100.0%)	0	
		No		96	71 (74.0%)	25 (26.0%)	

* Statistically significant difference was noted. CRG-carbapenem resistance genes. OPD, outpatient department; ICU, intensive care unit.

**Table 3:** Characteristics of patients who died during hospital stay and after hospital discharge.

Period of Death	Gender	Age (Years)	CRO Present	CRG Present
During hospital stay (*n* = 4)	Female	45	*K. pneumoniae*	None
	Male	49	*A. baumannii*	*bla*_OXA-51_ + *bla*_OXA-23_
	Female	69	*K. pneumoniae*	*bla*_OXA-48_
	Female	24	*K. pneumoniae*	*bla*_OXA-51_
Within 30 days of discharge (*n* = 4)	Male	65	*P. aeruginosa*	None
	Male	79	*S. marcescens*	*bla*_OXA-51_
	Female	80	*K. pneumoniae*	None
	Female	80	*K. pneumoniae*	*bla*_OXA-51_
Within 30–60 days of discharge (*n* = 1)	Male	82	*A. baumannii/calcoaceticus* complex	*bla*_KPC_ + *bla*_OXA-51_ + *bla*_OXA-58_
Within 60–90 days of discharge (*n* = 1)	Female	70	*E. coli*	*bla*_OXA-51_ + *bla*_OXA-58_

CRO; carbapenem-resistant organism, CRG; carbapenem-resistant gene.

## Data Availability

All the data generated and analyzed during this study are included in this manuscript. Further inquiries can be directed to the corresponding author.

## Abbreviations

The following abbreviations are used in this manuscript:

ABST	Antibiotic susceptibility testing
BPH	Benign prostatic hyperplasia
CFU	Colony-forming units
CRG	Carbapenem-resistant genes
CRO	Carbapenem-resistant organisms
CRE	Carbapenem-resistant enterobacteriaceae
CLSI	Clinical Laboratory Standard Institute
GNB	Gram-negative bacteria
ICU	Intensive care unit
MDR	Multidrug-resistant
MIC	Minimum inhibitory concentration
NCI	National Cancer Institute
OPD	Out-patient department
PCR	Polymerase chain reaction
UHKDU	University Hospital, Kotelawala Defence University
USA	United States of America
UTI	Urinary tract infection
WBC	White blood cells
WHO	World Health Organization
*bla*_KPC_	*Klebsiella pneumoniae* carbapenemase
*bla*_IMP_	Imipenemase
*bla*_NDM_	New Delhi metallo-β-lactamase
*Bla_OXA_*	Oxacillinase
*bla_V_*_IM_	Verona Integron encoding metallo-β-lactamase
